# Control of immune cell signaling by the immuno-metabolite itaconate

**DOI:** 10.3389/fimmu.2024.1352165

**Published:** 2024-02-28

**Authors:** Roland Lang, Md Nur A Alam Siddique

**Affiliations:** ^1^ Institute of Clinical Microbiology, Immunology and Hygiene, Universitätsklinikum Erlangen, Friedrich-Alexander-Universität Erlangen-Nürnberg, Erlangen, Germany; ^2^ FAU Profile Center Immunomedicine (FAU I-MED), Friedrich-Alexander-Universität (FAU) Erlangen-Nürnberg, Erlangen, Germany

**Keywords:** itaconate, IRG1, ACOD1, metabolism, macrophages, signal transduction, post-translational modification, transport

## Abstract

Immune cell activation triggers signaling cascades leading to transcriptional reprogramming, but also strongly impacts on the cell’s metabolic activity to provide energy and biomolecules for inflammatory and proliferative responses. Macrophages activated by microbial pathogen-associated molecular patterns and cytokines upregulate expression of the enzyme ACOD1 that generates the immune-metabolite itaconate by decarboxylation of the TCA cycle metabolite cis-aconitate. Itaconate has anti-microbial as well as immunomodulatory activities, which makes it attractive as endogenous effector metabolite fighting infection and restraining inflammation. Here, we first summarize the pathways and stimuli inducing ACOD1 expression in macrophages. The focus of the review then lies on the mechanisms by which itaconate, and its synthetic derivatives and endogenous isomers, modulate immune cell signaling and metabolic pathways. Multiple targets have been revealed, from inhibition of enzymes to the post-translational modification of many proteins at cysteine or lysine residues. The modulation of signaling proteins like STING, SYK, JAK1, RIPK3 and KEAP1, transcription regulators (e.g. Tet2, TFEB) and inflammasome components (NLRP3, GSDMD) provides a biochemical basis for the immune-regulatory effects of the ACOD1-itaconate pathway. While the field has intensely studied control of macrophages by itaconate in infection and inflammation models, neutrophils have now entered the scene as producers and cellular targets of itaconate. Furthermore, regulation of adaptive immune responses by endogenous itaconate, as well as by exogenously added itaconate and derivatives, can be mediated by direct and indirect effects on T cells and antigen-presenting cells, respectively. Taken together, research in ACOD1-itaconate to date has revealed its relevance in diverse immune cell signaling pathways, which now provides opportunities for potential therapeutic or preventive manipulation of host defense and inflammation.

## 1 Introduction

In the last decade, immunologists have realized that activation of innate and adaptive immune cells by pattern recognition and antigen receptors leads to decisive changes in the central metabolic processes, which in turn play an essential role in provision of the energy and the biomolecules required for immune cell proliferation, secretion of cytokines and chemokines, and generation of anti-microbial responses (1, 2). It has become clear that the central pathways of ATP generation, glycolysis and the tricarboxylic acid (TCA) cycle feeding oxidative phosphorylation in the mitochondria, are not a static machinery, but instead are regulated in distinct ways depending on the cell type and the stimulus. In addition, it is now established that glycolysis and mitochondrial TCA cycle not only produce the energy required for immune responses but that many metabolites generated by these pathways have important functions in immune cells, and are therefore referred to as immuno-metabolites (3). Prominent examples of such metabolites with immunological function are lactate (4), the end product of anaerobic glycolysis, the TCA cycle intermediates citrate (5), α-ketoglutarate and succinate (6).

However, the most prominent immune-metabolite is probably the dicarboxylic acid itaconate that is generated by the enzyme aconitate decarboxylase 1 (ACOD1) from the TCA intermediate cis-aconitate (7). Itaconate has only been identified as a relevant metabolite produced in macrophages in 2011 (8), followed by the identification of the gene *Acod1/Irg1* encoding ACOD1 as the enzyme generating it in 2013 (9). Itaconate was initially characterized as anti-microbial compound that can inhibit the growth of *Mycobacterium tuberculosis* (9) and of *Legionella pneumophila* (10). The anti-microbial activity of itaconate has been extended to several other bacteria, e.g. the intracellular pathogens *Salmonella typhimurium* (11), *Brucella* spp (12, 13). *Francisella tularensis* (14), *Mycobacterium avium* (15) and *Coxiella burnetii* (16), suggesting that its generation is an important mechanism of host defense.

On the other hand, immunoregulatory effects of itaconate were first described in 2016 as reduced production of several LPS-induced cytokines (IL-6, IL-12 and IL-1β) in macrophages treated with exogenous itaconate, and increased cytokine levels in macrophages unable to produce endogenous itaconate (17). These findings triggered intense research activity in many labs with a plethora of publications identifying roles for itaconate in innate and adaptive immune cell regulation and different mechanisms (from enzymatic inhibition to post-translational modification (PTM) of diverse target proteins) underlying these effects. While the diverse findings from the ACOD1-itaconate field are complex, the dual role of this metabolite as endogenous immunoregulatory and anti-microbial agent indicates a potential to develop itaconate or its synthetic derivatives for the treatment of acute and chronic inflammatory and infectious disease conditions.

In this review, we will first briefly summarize the stimuli and pathways controlling the inducible expression of the enzyme ACOD1 in innate immune cells. We will then focus on the impact of itaconate on immune cell signaling and metabolism through direct inhibition of several enzymes and *via* the PTM of target proteins, mostly at cysteine residues. The growing number of signaling proteins modified and regulated by itaconate can explain at the molecular levels many of the complex phenotypes observed by addition of exogenous itaconate or in cells deficient in ACOD1. The effects of itaconate on the function of immune cells in inflammatory responses and in infection are the topic of the next chapter, starting with innate immune cells (macrophages and neutrophils), and followed by the review of the less abundant literature on the regulation of adaptive immune responses by itaconate. Finally, we will conclude with a summary of what we see as the most important open questions in itaconate biology.

## 2 Generation of itaconate by ACOD1

### 2.1 Induction of ACOD1 expression

**Figure 1:**
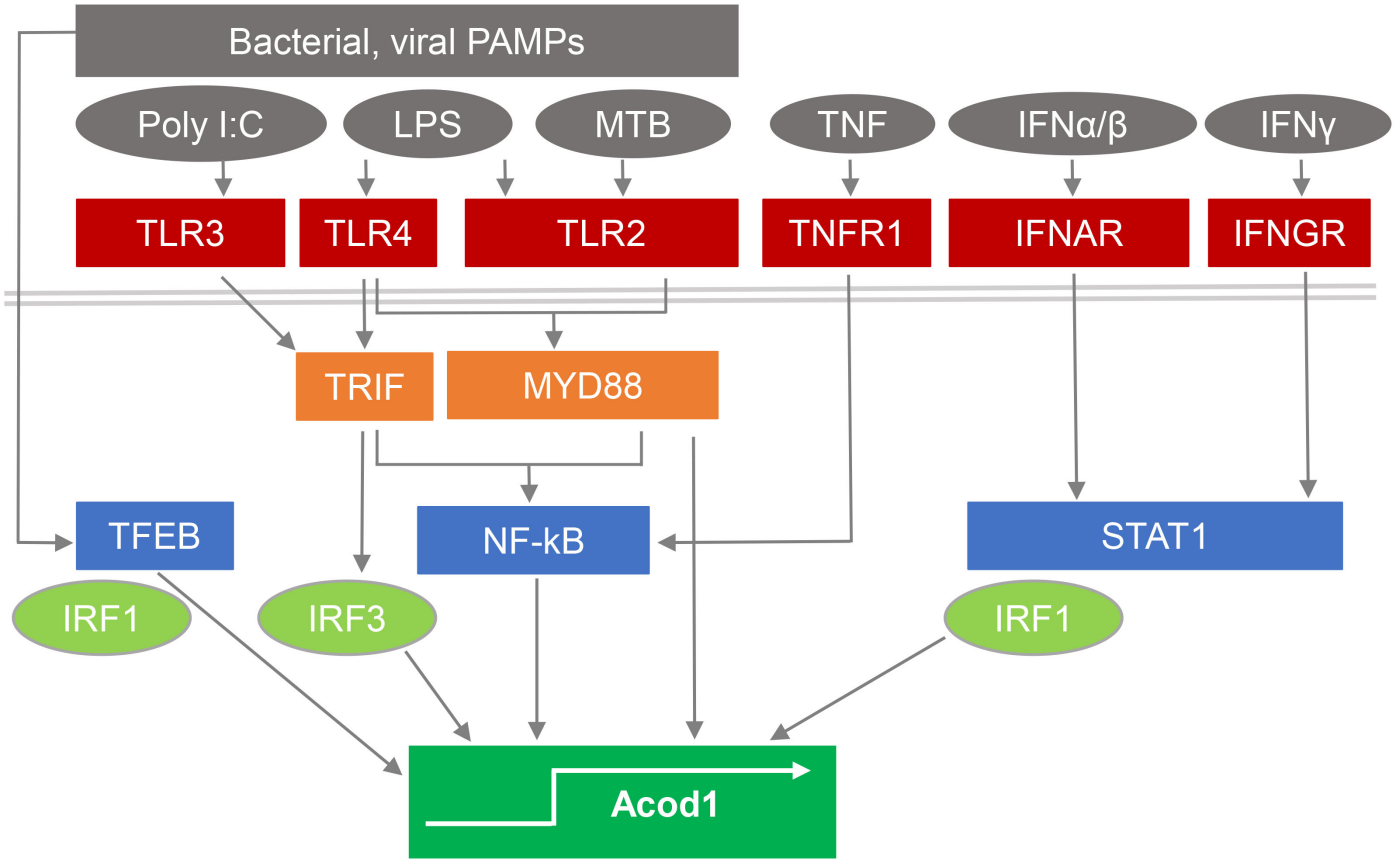
Pathways stimulating ACOD1 expression and itaconate production. Expression of ACOD1 is very low under basal conditions but rapidly upregulated in response to microbial PAMPs by activation of TLRs and signaling via TRIF and MyD88. *M. tuberculosis* (MTB) induces ACOD1 through TLR2/MyD88 but also via STING activation and IFNβ production (25). Translocation of TFEB to the nucleus in response to microbial stimulation is sufficient to increase ACOD1 mRNA in a partially IRF1-dependent manner (26). TNF and interferons induce ACOD1 expression through NFκB- and STAT1-dependent pathways and can act in synergy (23).

The gene encoding ACOD1, formerly named *Irg1*, has popped up in screens for IFNγ- and TLR-induced macrophage or dendritic cell (DC) mRNAs even before the introduction of microarrays (18, 19), and was confirmed as a highly induced gene by many studies on the response to macrophage stimulation by bacterial PAMPs or infection (16, 20–22), or activation with type I IFN (10), IFNγ and/or TNF (23, 24) (see **Figure 1**). High level induction of *Acod1*/*Irg1* mRNA in response to bacterial PAMPs required the TLR adapter proteins MyD88 (27) and TRIF (for the TLR3/4 ligands polyI:C and LPS) (21), whereas the response to IFNs was dependent on the transcription factors STAT1 (20) and IRF1 (28). In response to *M. tuberculosis* (MTB), ACOD1 expression depended on the TLR2-MyD88 pathway as well as on the ESX1-triggered activation of a STING-IFNAR1-dependent response (25). The transcription factor TFEB, a key regulator of lysosomal biogenesis (29), translocates to the nucleus after stimulation with LPS/IFNγ, MTB or with *Salmonella typhimurium* and is sufficient to increase ACOD1 mRNA in a partially IRF1-dependent manner (26).

More recently, also neutrophils have been identified to upregulate ACOD1 mRNA expression to high levels in infection and tumor models (30, 31). In contrast, adaptive immune cells have not been described to express Acod1 or to produce significant amounts of itaconate.

### 2.2 Mitochondrial localization of ACOD1 and itaconate

**Figure 2:**
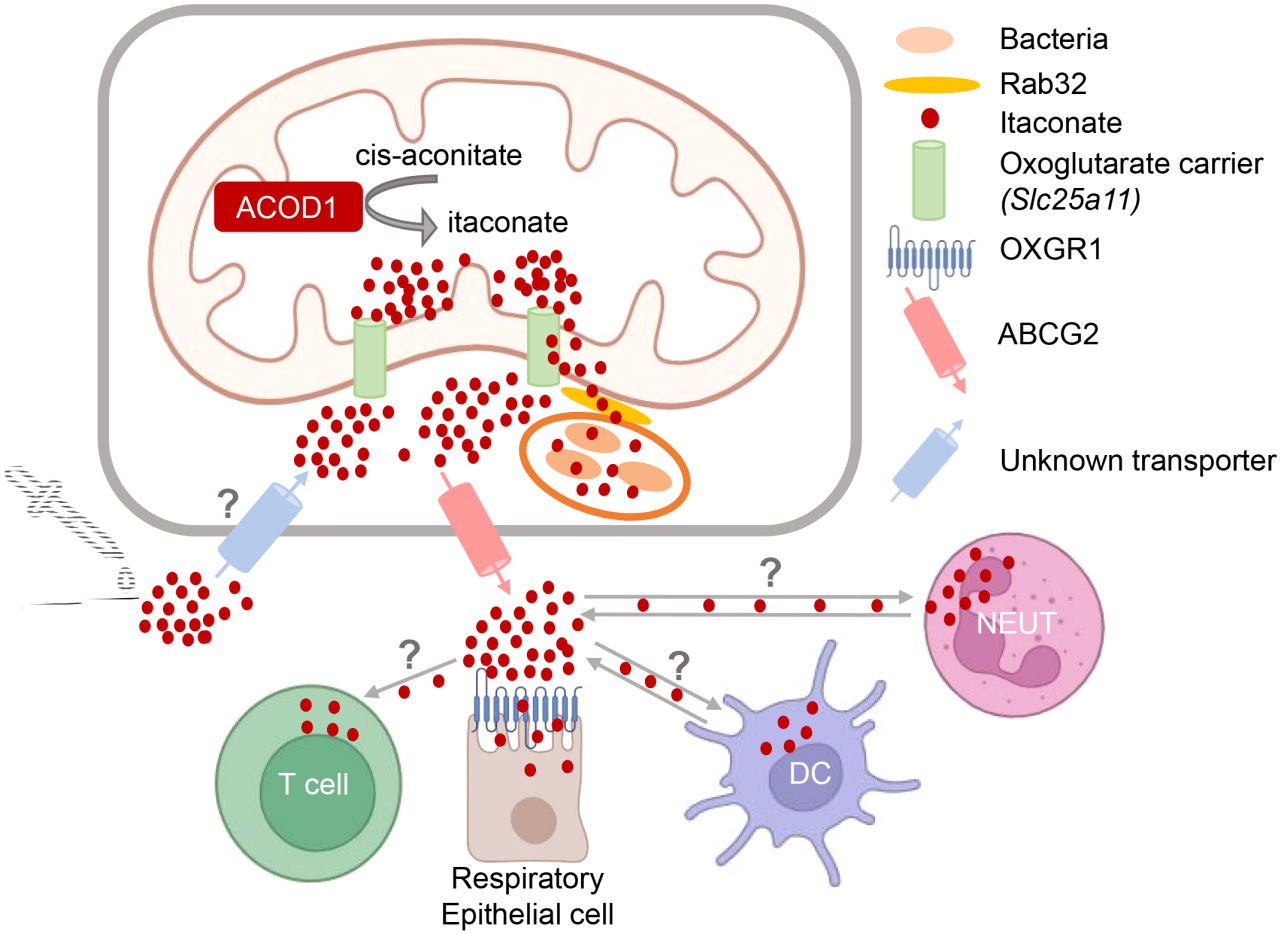
Intra- and intercellular trafficking of itaconate: knowns and unknowns. Itaconate is generated in the mitochondria and presumably transported out into the cytoplasm by the oxoglutarate carrier OGC (35). Transfer of itaconate to bacteria-containing phagosomes requires Rab32 and appears to be facilitated by the close proximity of mitochondria and vacuoles containing *Legionella* (10) or *Salmonella* (11, 36). How itaconate passes the phagosomal membrane to reach intravacuolar bacteria is unknown. Putative transporters for uptake of exogenous itaconate (and its derivatives) and for release of endogenous itaconate into the extracellular space are indicated. T cells do not produce itaconate but can take it up (37). Activated neutrophils and DC can both express itaconate (21, 30, 31, 38), but may also take it up from the microenvironment. Itaconate can bind to OXGR1 receptor on respiratory epithelial cells (39).

ACOD1 protein is localized to the mitochondria (10, 23), where the TCA cycle is operating. ACOD1 catalyzes the conversion of cis-aconitate, derived from citrate, by decarboxylation to itaconate. In resting macrophages itaconate is not detectable, consistent with the low basal expression of ACOD1. In contrast, within hours after stimulation with LPS, the rapid induction of ACOD1 protein results in production of itaconate that is detectable intracellularly at millimolar concentration in mouse macrophages (9, 11, 32). The essential and non-redundant function of ACOD1 in generation of itaconate was demonstrated in macrophages from *Acod1^-/-^* mice (17). Within the mitochondria, itaconate competes with succinate for binding to succinate dehydrogenase (SDH) and inhibits its enzymatic activity, causing a break in the TCA cycle and accumulation of succinate (6, 33). Itaconate can then be further metabolized to Itaconyl-CoA (34). Itaconate is transported out of the mitochondria and into the cytoplasm (**Figure 2**), presumably by the 2-oxoglutarate carrier (OGC, encoded by *Slc25a11*), and to a lesser extent by the citrate carrier (CTP, encoded by *Slc25a1*) and the dicarboxylate carrier (DIC, encoded by *Slc25a10*) (35), where it exerts some of its immunoregulatory activities by binding to signaling proteins and transcription factors (discussed in detail below).

### 2.3 Trafficking of itaconate

The intra- and inter-cellular trafficking of itaconate is only beginning to be elucidated (**Figure 2**). This research area is of high relevance for our understanding how itaconate acts during immune responses and for the potential therapeutic modulation of itaconate levels. Since itaconate is a polar molecule, passive diffusion via cellular membranes is unlikely. The anti-microbial activity of itaconate against intracellular bacteria within macrophages requires its delivery to the phagolysosomal compartments containing pathogens like MTB, *C. burnetii*, *Salmonella* spp. or *Legionella pneumophila*. Work by the Galan lab has shown that transport of itaconate produced in the mitochondria to *Salmonella*-containing vacuoles is dependent on Rab32-mediated GTPase activity (11), with the Parkinson’s disease-associated leucine-rich repeat kinase 2 (LRRK2) acting as a scaffold bringing mitochondrial ACOD1 into complex with Rab32 and tethering mitochondria to the pathogen-containing phagosome (36). However, how itaconate then passes the phagosomal membrane remains to be established. Similarly, it is clear that itaconate is released by the producing cells into the extracellular space, as it is detected in considerable amounts in the cell culture supernatants of activated macrophages (39); however, whether this is a transporter-dependent process and which proteins are involved in the secretion from cells is only beginning to be elucidated. Very recently, ATP-binding cassette transporter G2 (ABCG2) was identified in a genetic screen as mediating the active release of itaconate from human and mouse macrophages (40). Finally, while the notion that the polar nature of itaconate would prevent its intracellular penetration when given exogenously (35) has sparked the use of dimethyl-itaconate (DMI) and 4-octyl-itaconate (4-OI) in many studies, it is now clear that itaconate from the extracellular space is readily taken up by macrophages (15, 16, 41). To date, the only known cell surface receptor for itaconate is the oxoglutarate receptor OXGR1 on respiratory epithelial cells, a GPCR whose ligation with itaconate leads to Ca^2+^ mobilization, mucus secretion and transport (39).

### 2.4 Derivatives and isomers of itaconate

When employing the synthetic derivatives of itaconate, especially 4-OI and DMI, it is important to keep in mind that they are not converted to itaconate in macrophages (41, 42), although this may be different for 4-OI (43), and may have distinct effects due to their stronger electrophilic properties (41), see also below (**Table 1**). Thus, some of the findings made using 4-OI or DMI in early papers, e.g. the strong Nrf2-dependent regulation of cytokine production (35), need to be re-interpreted with caution.

**Table 1:** Itaconate, its isomers and derivatives.

Molecule	Structures	Source	Electro-philicity	Nrf2 activator	Intracellular conversion to itaconate
Itaconate	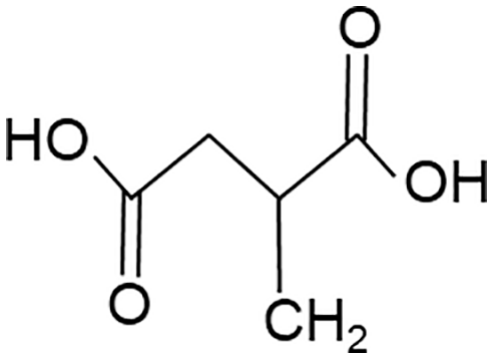	ACOD1	+	+/-	Yes
4-Octyl Itaconate (4-OI)	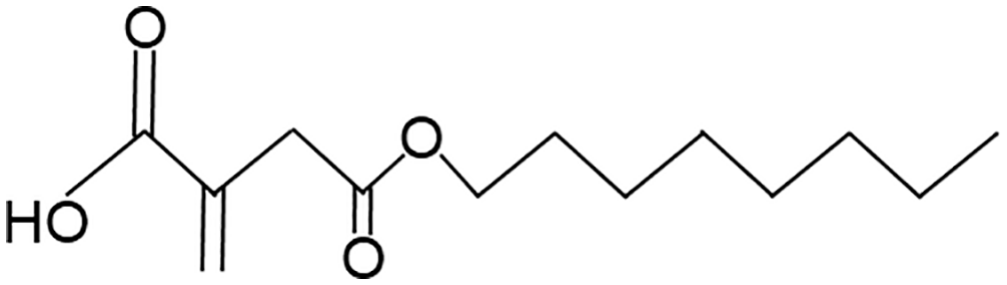	Chemically synthesized	+++	+++	Yes (ref. 42) No (ref. 40)
Dimethyl Itaconate (DMI)	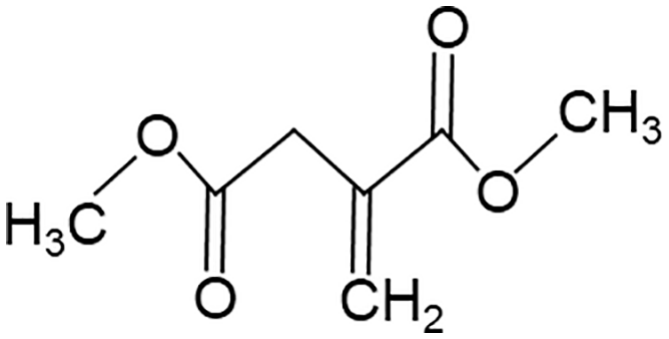	Chemically synthesized	++	+++	No
Mesaconate	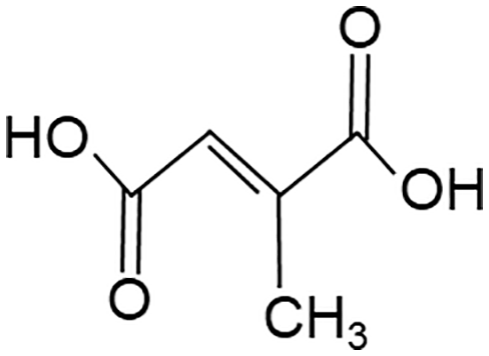	ACOD1	(+)	(+)	No
Citraconate	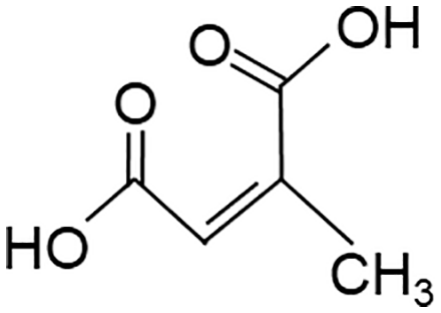	Not made by macrophages	+++	+++	Inhibits ACOD1

An interesting extension of ACOD1 activity was introduced by the demonstration in 2022 that in addition to itaconate its isomer mesaconate is also generated in activated macrophages in an ACOD1-dependent manner from itaconate (44). The endogenous levels of mesaconate are significantly lower compared to itaconate (in the range of 1 to 10%). Exogenously added mesaconate showed similar immunoregulatory activity in terms of cytokine production, but was less inhibitory for SDH and glycolysis compared to itaconate. At the same time, another report described citraconate as an isomer of itaconate that was not generated endogenously in macrophages but was readily taken up by them (45). Citraconate acted as the strongest electrophilic agent and reduced cytokine production (45). Interestingly, citraconate inhibited ACOD1 enzymatic activity *in vitro* and in cells (45), and hence may become of interest to develop pharmacologically.

## 3 Impact of itaconate on cell signaling and metabolism

**Figure 3:**
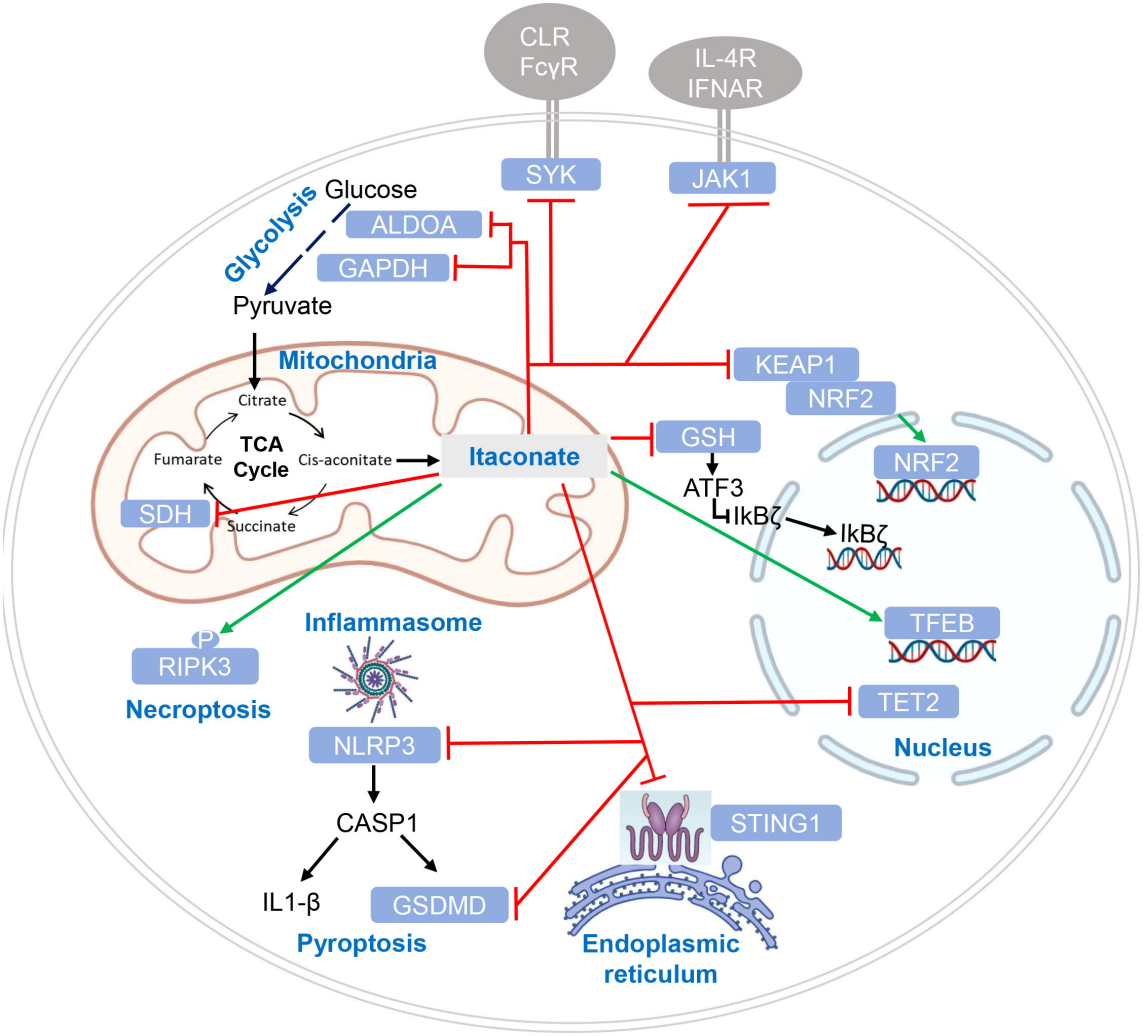
Targets of itaconate regulation by enzymatic inhibition and post-translational modification. Target proteins of itaconate and its derivatives are indicated in white letters within light blue boxes. Red lines indicate inhibitory effects of itaconate on protein function, green lines indicate enhanced function or protein levels. Cellular compartments are indicated where modification or inhibition of target proteins occurs. CLR (C-type lectin receptors) and FcγR (Fc gamma receptors) are shown as SYK-coupled receptors putatively affected by itaconation of SYK (46); cytokine receptors in addition to IL-4 receptor and IFNAR may be affected by itaconate (47), given the pleiotropic function of JAK1.

Itaconate and its derivatives exert numerous effects on the activating signaling pathways leading to cytokine production, inflammation and the response to infection in macrophages (**Figure 3**). Mechanistically, these effects can be divided into three categories: (1) activation of an anti-oxidant response to electrophilic stress, (2) inhibition of enzymatic activity of SDH and of Tet2, and (3) itaconate-mediated PTM of signaling proteins leading to functional alterations.

### 3.1 Electrophilic stress, glutathione buffering and ATF3 inhibition of IκBζ

The tripeptide glutathione is an important cellular anti-oxidant buffer system, shuttling electrons between its reduced GSH and oxidized GSSG form (48). Treatment of macrophages with DMI led to the formation of a diester of methylsuccinated GSH through Michael addition, and the same reaction occurred with endogenous itaconate in LPS-activated macrophages (49). A role for this depletion of the GSH antioxidant buffer system in the inhibition of cytokine production by DMI was suggested by the restoration of IL-6 production after addition of the antioxidant *N*-acetylcysteine (NAC). Inhibition of the secondary LPS response gene *Il6* but not of *Tnf* by DMI pointed to a role for IκBζ, whose upregulation was indeed repressed by DMI and restored by NAC. The transcription factor ATF3, a component of the integrated stress response, was induced by DI and in an ACOD1-dependent manner in LPS-tolerized macrophages, and was required for repression of IκB*ζ* expression and inhibition of IL-6 production by DMI (49). Later work using itaconate and its isomer mesaconate showed that both inhibit LPS-induced IL-6 independent of ATF3 (44), suggesting that the more electrophilic DMI has a higher propensity to disrupt GSH buffering and to induce ATF3-dependent inhibition of cytokine expression.

### 3.2 Enzymatic inhibition

The analysis of *Acod1^-/-^* macrophages revealed that all LPS-induced itaconate production was completely abrogated (17, 33). At the same time, the increase in succinate levels observed in stimulated WT macrophages was also normalized, indicating that itaconate inhibits the TCA cycle enzyme succinate dehydrogenase (SDH) (17, 33), which was confirmed with purified enzyme (17). As SDH also forms complex II of the respiratory chain, its inhibition by itaconate leads to a reduction in the mitochondrial oxygen consumption rate (OCR) in LPS-activated macrophages (17, 33). Consequently, *Acod1^-/-^* macrophages stimulated with LPS did not show the reduction in OCR observed in WT macrophages, but in fact an increased OCR (17).

Tet2 is a ketoglutarate-dependent DNA dioxygenase that epigenetically impacts on gene expression by converting 5-methylcytosine to 5-hydroxymethylcytosine, which promotes demethylation (50). Tet2 inactivation reduces the LPS-induced expression of many cytokines and chemokines. Tet2 is competitively inhibited by several ligands structurally similar to α-ketoglutarate (succinate, fumarate and 2-D-hydroxyglutarate). Itaconate inhibits Tet2 activity by competing with the binding of α-ketoglutarate (43). The majority of LPS-induced genes that are downregulated by 4-OI are Tet2-dependent, indicating that a significant part of the immunoregulatory itaconate effect is caused by inhibition of Tet2 (43). This notion is mechanistically supported by the finding that recruitment of Tet2 to the *Nfkbiz* promoter after LPS is mediated by binding to the NFκB family member RelA (43).

### 3.3 Protein modification by itaconate

The accumulation of the TCA cycle metabolites succinate, fumarate and lactate during the metabolic shifts occurring in macrophages in response to pathogen stimulation is accompanied by PTM of proteins by these carboxylic acids, e.g. by lysine acylation by succinyl-CoA (51) or lactyl-CoA (52), as well as by Michael addition of cysteines by fumarate (53). The dicarboxylic acid itaconate and its derivatives 4-OI and DMI can also react by Michael addition with cysteines to form a 2,3-dicarboxypropyl adduct, a process now termed itaconation. This formation of a thioether bond appears to be non-reversible, generating a stable PTM of the itaconated protein until it is degraded. The discovery of itaconated proteins has been driven by the development of an alkyne-modified probe built on the structure of 4-OI (ITalk), that is cell permeable but not hydrolyzed in macrophages, to perform a systematic chemoproteomic screen for this PTM in cells (42). Nearly 2000 itaconated proteins were identified by using the ITalk probe in the RAW264.7 macrophages cell line, suggesting that this PTM is highly abundant in activated macrophages. Itaconated proteins included previously described targets like KEAP1 and LDHA (see below), and many key proteins involved in inflammatory response and host defense pathways. Several of these were validated in the same study, e.g. the receptor-interacting serine/threonine kinase 3 (RIPK3) that is essential for necroptosis and whose phosphorylation was enhanced by itaconate dependent on one of the itaconated cysteines (42). While RIPK3 is an example for a protein where itaconation is required for its function, the enzymatic activity or protein-protein interactions of other target proteins are inhibited by this PTM (54).

In addition to itaconation by formation of a thioether at cysteine residues, very recently the itaconate-mediated lysine acylation was discovered as a new type of PTM, requiring the prior synthesis of itaconyl-CoA and termed “itaconylation” (55). Itaconylation was found on several glycolytic enzymes (GAPDH, ENO1, PKM2 and LDHA), histone H2B1B, Actin, and others, with 87 itaconylation sites identified in LPS-activated RAW 264.7 macrophages in total (55). Interestingly, itaconyl-CoA was only produced in RAW 264.7 but not in several other cancer cell lines, indicating that itaconylation may particularly affect immune responses. Furthermore, treatment with 4-OI does not cause itaconylation because it lacks a critical carboxylate group for formation of itaconyl-CoA (55), which may underly some of the different effects of itaconate and 4-OI on immune cells.

#### 3.3.1 KEAP1

The first identified target protein of itaconate was Kelch-like ECH associated protein (KEAP1) (35) that binds to the regulatory transcription factor Nrf2 and promotes its ubiquitin-mediated proteasomal degradation. Itaconation of KEAP1 prevents its binding to Nrf2, protecting newly synthesized Nrf2, allowing it to accumulate and enabling its nuclear translocation (35). Nrf2 is a key transcription factor of the response to oxidative stress and regulates metabolic pathways (56). The immunoregulatory effect of 4-OI, measured as reduction in pro-IL-1β was strongly dependent on Nrf2 (35). Endogenous itaconate was required to increase Nrf2 protein in LPS-stimulated macrophages (49). Induction of the Nrf2 response by different itaconate derivatives correlates with their electrophilicity, with the highly electrophilic derivatives 4-OI and DMI inducing more Nrf2-dependent gene expression than itaconate (41). Similarly, the isomer citraconate is more electrophilic than itaconate and mesaconate, and induces more Nrf2 and Nrf2-dependent gene expression (45) (**Table 1**).

Interestingly, KEAP1 can also be modified at cysteine residues by fumarate and glyceraldehyde 3-phosphate (57, 58), and by succinylation at a critical lysine sensor residue, also leading to increases in Nrf2 protein levels (59). Hence, it appears that these PTMs of KEAP1 serve as a generic switch to induce a regulatory stress response to different reactive metabolites (59).

#### 3.3.2 GAPDH

Glyceraldehyde 3-phosphate dehydrogenase (GAPDH) is a rate-limiting enzyme in aerobic glycolysis and contributes to the glycolytic shift occurring in macrophages after TLR-stimulation. GAPDH was found to be itaconated at Cys22 in LPS-activated or 4-OI-treated RAW 264.7 macrophages (60). Consistent with inhibition of GAPDH by itaconation at Cys22, treatment of LPS-stimulated macrophages with 4-OI inhibited generation of lactate and reduced the increase in the extracellular acidification rate (ECAR), whereas *Acod1^-/-^* BMDM showed the opposite effect (60). Treatment with the GAPDH-inhibitor heptelidic acid inhibited production of TNF, IL-1β and expression of iNOS similarly as 4-OI. The impact of glycolysis on LPS-induced cytokine production was shown by use of the hexokinase inhibitor 2-DG and by the attenuation of the inhibitory 4-OI effect in macrophages cultured in high (25 mM) vs. low (0.5 mM) glucose concentration. Overexpression of GAPDH, but not a GAPDH-Cys22Ala mutant, enhanced IL-1β production by LPS-activated macrophages (60).

#### 3.3.3 ALDOA and LDHA

Chemoproteomic profiling revealed that itaconate modifies not only GAPDH on cysteines but also two other key enzymes important for glycolysis, namely fructose-biphosphate aldolase A (ALDOA) and L-lactate dehydrogenase A (LDHA) (61). Itaconate inhibited ALDOA activity and reduced glucose consumption and lactate production (61). Together, the studies showing itaconation of key enzymes of aerobic glycolysis and inhibition of their activity by itaconate and 4-OI demonstrate a counter-regulatory mechanism at the level of glucose metabolism for the inducible production of itaconate in TLR-activated macrophages: the shift to glycolysis is attenuated, which prevents over-production of several inflammatory cytokines (e.g. IL-1β).

#### 3.3.4 NLRP3

The NLRP3 inflammasome is an important sensor of pathogenic invasion and endogenous cellular damage. It is composed of the sensor protein NLRP3, the adaptor protein ASC and the enzyme caspase-1 which processes IL-1β, IL-18 and gasdermin D (GSDMD). The observation of increased IL-1β secretion by *Acod1^-/-^* macrophages (17) suggested that regulation of the inflammasome may be one of the mechanisms of immunoregulation by itaconate. Indeed, NLRP3 was identified as an itaconated protein in the screen performed by Qin et al. in 2020 (42), followed by the demonstration that itaconation of NLRP3 on Cys548 interferes with its binding to NEK7 (62), which is required for efficient formation of the NLRP3 inflammasome disc and of the NLRP3 pyrin domain filament (63). This inhibition was specific for the NLRP3 inflammasome, as IL-1β production induced by stimuli triggering the NLRC4 and AIM2 inflammasomes was not affected by 4-OI and not enhanced in *Acod1^-/-^* macrophages (62).

#### 3.3.5 GSDMD

Among the substrates for caspase-1 activated by the NLRP3 inflammasome are not only the pro-forms of IL-1β and IL-18 but also the pore-forming protein Gasdermin D (GSDMD). The N-terminal fragments of cleaved GSDMD assemble into the membrane pore required for secretion of IL-1β and occurrence of pyroptotic cell death (63). Again, itaconation of GSDMD was first found in the Qin et al. dataset (42), and the modification of GSDMD Cys77 was linked to the reduced production of IL-1β through the demonstration that caspase-1 processing of GSDMD is blocked in LPS-tolerized macrophages in an ACOD1-dependent manner (64). The impact of ACOD1-derived endogenous itaconate is limited to tolerance induction for late inflammasome activation 24 hours after LPS stimulation (64). Interestingly, full activation of caspase-1 late after LPS was abrogated in *Gsdmd^-/-^* macrophages (64). Together, it appears that itaconate inhibits both early and late inflammasomes to reduce IL-1β secretion through distinct effects of itaconation of NLRP3 and GSDMD, although it is important to keep in mind that the use of highly electrophilic derivatives of itaconate (such as 4-OI) complicates the interpretation of the results.

#### 3.3.6 JAK1

Runtsch et al. examined the question whether itaconate (or 4-OI) affects the response of macrophages to stimulation with the Th2 cytokine IL-4 (47). Indeed, 4-OI and itaconate dose-dependently inhibited the induction of several genes typical for IL-4 polarized M2 macrophages (e.g. FIZZ1 and PPARγ). Phosphorylation of the IL-4 triggered transcription factor STAT6 was reduced by itaconate and 4-OI, which hinted to inhibition of the IL-4 receptor-associated kinase JAK1. Indeed, JAK1 was inhibited by itaconate and 4-OI at relevant concentrations (although less efficiently than by pharmacological JAK1 inhibitors). Treatment of transfected HE293T cells with the ITalk probe led to itaconation of overexpressed JAK1 at four cysteine residues. These findings were confirmed in human THP-1 macrophages treated with 4-OI. Treatment of mice with 4-OI *in vivo* reduced M2 gene expression after IL-4-complex injection in peritoneal exudate cells, and ameliorated symptoms in a Th2-driven asthma model (47). Most of the data shown in the paper were obtained using 4-OI, not itaconate, and since IL-4 does not trigger expression of ACOD1 and itaconate production, no experiments were shown using *Acod1^-/-^* mice or macrophages. However, during immune responses *in vivo*, especially in certain infections, ACOD1 expression and itaconate production will occur concomitantly with IL-4 secretion (e.g. by ILC2 and Th2 cells). It will be of interest to test whether endogenous itaconate affects IL-4 receptor-driven polarization of macrophages and T cells under these conditions.

#### 3.3.7 SYK

Spleen tyrosine kinase (SYK) is recruited to phosphorylated immunoreceptor based activation motifs in the intracellular domains of cell membrane localized receptors or their adapter proteins (65). SYK plays an essential role in the signaling of the B cell receptor, activating Fc receptors and myeloid C-type lectin receptors like Dectin-1 and Mincle (65). In a mouse model of infection with hypervirulent *Klebsiella pneumoniae* (hvKP), Li et al. observed strong SYK activation in macrophages that was coupled to expression of ACOD1 and production of itaconate (46). Exogenous itaconate, 4-OI or DMI reduced cytokine production and SYK activation in macrophages exposed to hvKP, whereas enhanced phosphorylation of SYK occurred in *Acod1^-/-^* macrophages. SYK was found to be itaconated at cysteines 587 and 591 (mouse), corresponding to Cys593 and Cys597 in human SYK, which are located in the kinase domain. Mutation of Cys593 in human SYK abrogated inhibition by itaconate and its derivatives (46). Administration of itaconate or 4-OI to mice infected with hvKP reduced inflammation in the gut, weight loss and mortality, whereas *Acod1^-/-^* mice showed a more severe phenotype (46). It will be interesting to see whether other, well characterized SYK-activating receptors in innate immune cells, e.g. Dectin-1 triggered by Curdlan, or Mincle by the mycobacterial cord factor, induce a similar itaconate-driven negative feedback loop via itaconation of SYK kinase.

#### 3.3.8 STING

Stimulator of interferon genes (STING) is an essential component of cytosolic DNA sensing. STING binds 2’,-3’-cGAMP generated by cGAS in response to dsDNA, or to bacterially produced c-di-AMP or c-di-GMP, and triggers activation of TBK1 and IRF3, leading to transcription of IFNβ and activation of NFκB signaling (66). In response to the STING activator DMXAA, macrophages upregulate ACOD1 expression and generate itaconate in a TBK1- and IFNAR1-dependent manner (67). Itaconate and 4-OI, but not citraconate, citrate or α-ketoglutarate, inhibited phosphorylation of STING, TBK1 and IRF3, as well as expression of IFNβ, IL-1β and IL-6 (67). Using LC-MS/MS of HEK293T cells overexpressing STING and treated with 4-OI, itaconation of STING at four cysteine residues was detected. Analysis of mutants in the individual cysteines transfected into RAW 264.7 cells showed that Cys88 and Cys147 are functionally important for induction of IRF3 activation and IFNβ expression. Whereas reduced phosphorylation of the STING Cys88Ser mutant was further inhibited by 4-OI, the mutation in Cys147 affected the dimerization of STING that is also inhibited by 4-OI (67). In an *in vivo* model of septic peritonitis (CLP), *Acod1^-/-^* mice had higher mortality, with increased TBK1 activation and IFNβ expression in intestinal tissues, whereas exogenous ITA or 4-OI administration increased survival. A direct involvement of STING modifications by itaconate was not shown *in vivo*, but STING knockout mice did show decreased TBK1 activation and IFNβ expression after CLP, suggesting that there may be a causal relationship.

#### 3.3.9 TFEB

Transcription factor EB (TFEB) belongs to the microphthalmia family of basic helix-loop-helix leucine-zipper (bHLH-Zip) transcription factors proteins. TFEB is a master regulator of lysosomal biogenesis and function (29). Under nutrient-rich conditions it is phosphorylated by mTORC1 on Ser211, which is bound by the chaperone 14-3-3, leading to TFEB retention in the cytosol (29). Starvation, but also ER and mitochondrial stress, as well as TLR activation, lead to its nuclear translocation, binding to so-called CLEAR motifs in promoters of a network of genes involved in lysosomal biogenesis (29). Zhang et al. tested whether itaconate is involved in TFEB activation and observed that treatment of THP-1 cells and immortalized bone marrow-derived macrophages with 4-OI and itaconate was sufficient to cause nearly complete nuclear translocation of TFEB (68). Further, TFEB translocation driven by LPS or infection with *Salmonella typhimurium* was abrogated in *Acod1^-/-^* macrophages, but recovered by 4-OI (68). Itaconation of TFEB at Cys212 was detected by mass spectrometry analysis of THP-1 cells treated with 4-OI or itaconate and led to a loss of interaction with the 14-3-3 protein, nuclear translocation and enhanced expression of the lysosomal genes LAMP1, cathepsins B and D, and the proton pump ATP6V1H (68). All these effects were abrogated by introduction of a Cys212Ser mutation, suggesting that indeed the alkylation at this site is essential and acts by interfering with binding of 14-3-3 to phosphorylated TFEB. Finally, the corresponding mutation in murine TFEB at Cys270Ser was introduced by CRISPR/Cas9 in the mouse *Tfeb* locus, allowing to test the consequences for antibacterial defense *in vivo*. Following infection with *S. typhimurium*, TFEB C270S mice developed significantly higher bacterial burden and formed more microabscesses in the liver, and had a reduced survival time (68). Administration of 4-OI to infected wild-type mice reduced IL-1β and IL-18 in the serum, increased lysosomal staining from peritoneal macrophages, and prolonged survival after *S. typhimurium* infection. Together, alkylation of TFEB on Cys212 by itaconate promotes lysosomal biogenesis that enables infected cells to destroy invading bacteria.

A different perspective on the connection between itaconate and TFEB was introduced by the work of Schuster et al., demonstrating that overexpression of TFEB in macrophages is sufficient to induce expression of genes involved in glucose metabolism and the TCA cycle, including *Acod1/Irg1* (26). Production of itaconate induced by TFEB overexpression in resting macrophages was quantitatively comparable to the levels obtained after stimulation with LPS+IFNγ. Endogenous TFEB was required for full induction of itaconate production by bacterial stimulation of macrophages. TFEB induced ACOD1 mRNA expression independent of IFNAR1 and was only partially dependent on IRF1. The impact of TFEB on the macrophage capacity to deal with intracellular bacteria was also addressed in *Salmonella* infection. Treatment of mice with the pharmacological TFEB activator 2-hydroxypropyl-β-cyclodextrin (TFEBa) reduced the percentage of infected splenic macrophages in an ACOD1-dependent manner (26), suggesting that itaconate production is responsible for the anti-bacterial effect and consistent with a direct inhibition of *Salmonella* replication by itaconate (11). Looking at the studies by Zhang et al. and Schuster et al. in synthesis (26, 68), TFEB and itaconate seem connected in a mutually enhancing relationship to arm macrophages with sufficient lysosomal biomass and itaconate as anti-microbial strategies to fight intracellular bacteria.

## 4 Effects of itaconate on immune responses

### 4.1 Innate immune cells

#### 4.1.1 Macrophages

Expression of ACOD1 is restricted to myeloid cells, and macrophages are the main producers of itaconate, where it reaches intracellular concentrations in the millimolar level. The investigation of the effects of endogenous itaconate and of exogenously added derivatives has therefore focused on the regulation of macrophage activation and function.

Inhibitory activity of itaconate and its derivatives on production of pro-inflammatory cytokines by macrophages stimulated with TLR ligands was the first indication of the immunoregulatory function of itaconate (35, 49), and was corroborated in *Acod1^-/-^* macrophages that respond with higher IL-6, IL-12 and IL-1β secretion to LPS (17). Among the itaconate-regulated genes is also *Nos2* (17) that encodes the enzyme iNOS required for generation of nitric oxide (NO), a hallmark of M1 macrophage polarization (69). Thus, itaconate limits the M1 polarization of macrophages. *In vivo*, the impact of ACOD1-itaconate on the endotoxin-model of sepsis was in general beneficial, in that application of exogenous itaconate derivatives reduced pro-inflammatory cytokine levels in mice challenged with LPS (35, 44, 60, 70) and reduced lethality (35, 44, 60) or acute lung injury (70) in this model. The inhibitory effects on the inflammatory response to LPS are likely mediated by the combined action of itaconate on the mechanistic targets at the level of enzymatic inhibition (e.g. SDH, Tet2) and PTM by itaconation (e.g. GAPDH, ALDOA, KEAP1, SYK) and itaconylation described above in section 3. Beyond transcriptional regulation of gene expression, inhibition of IL-1β generation at the level of inflammasome activation through NLRP3 modification and NEK7 interaction (62, 64), as well as *via* modification of GSDMD (64), are important control points contributing to beneficial effects in sepsis and inflammation models (71, 72).

Exposure to PAMPs can result in priming, innate immune training or induction of tolerance to a secondary signal of infectious danger (73). The ACOD1-itaconate pathway can affect this decision in both directions, depending on the experimental setting. A role for ACOD1 in LPS tolerance was first described by Li et al. who found that ACOD1 enhanced expression of the anti-inflammatory gene TNFAIP3/A20 (74). Tolerance at the level of inflammasome activation is then again induced by itaconate-dependent interference with Caspase-1 activation and processing of GSDMD (64). The impact of stimulation with β-glucan, an inducer of trained immunity, on the development of tolerance was examined by Domingues-Andres et al. who found inhibition of LPS-induced ACOD1 expression by β-glucan in human monocytes, that was accompanied by a restoration of SDH expression and TCA cycle metabolites, and resulted in reversal of cytokine production (75). In a recent follow-up study, the same group reported that pre-treatment of human monocytes with DMI in a trained immunity-protocol increased cytokine production to secondary TLR stimulation (76). This effect was more pronounced for DMI than for itaconate, but still indicates that the ACOD1-itaconate axis can enhance innate immune responses under certain conditions. It remains to be understood which exact rules determine the consequences of ACOD1 expression and itaconate generation in up- or down-regulation of pro-inflammatory cytokines.

The regulation of interferon-dependent gene expression by itaconate and its derivatives is another example of complex phenotypes and partially conflicting reports. While 4-OI and DMI suppressed the interferon response to LPS (35), it was later demonstrated that itaconate itself rather promotes LPS-induced IFNβ secretion and *Acod1^-/-^* macrophages showed reduced expression of IFNβ and ISG responses (41). Similarly, He et al. observed enhanced expression of IFNβ and Cxcl10 in mouse macrophages treated with itaconate or mesaconate (44). In the context of immune activation via STING, e.g. after treatment with DMXAA, endogenous itaconate as well as 4-OI inhibited IFNβ expression through alkylation of STING and inhibition of its phosphorylation (67).

Finally, polarization of macrophages towards an M2 phenotype in response to IL-4 is reduced by exogenously added itaconate and 4-OI (47). This effect is caused by the inhibition of JAK1 activity through itaconation at several cysteines. The physiological significance of this finding is at present not clear because no data were shown for *Acod1^-/-^* mice or cells. However, 4-OI reduced M2 gene expression and airway resistance in a murine asthma model (47), indicating the potential for therapeutic modulation of IL-4-induced pathologies *in vivo*. On the other hand, the central role of JAK1 in the signaling not only of the IL-4 receptor but also in the response to type I interferon, and in fact several other type I and type II cytokine receptors (77), suggests a potentially highly pleiotropic effect of 4-OI and maybe also of endogenous itaconate on the response to cytokines.

#### 4.1.2 Neutrophils

As usual, innate immune researchers have neglected the role of neutrophils also in the investigation of ACOD1 and itaconate. However, this is changing recently, with several studies focusing on generation of itaconate by neutrophils and the impact of this metabolite on neutrophil numbers and function in different settings.

The strong phenotype of *Acod1^-/-^* mice in experimental infection with *M. tuberculosis*, with high bacterial burden and early death, was coupled to excessive accumulation of neutrophils in the lungs and the development of neutrophil-mediated immunopathology (78). Antibody-mediated depletion of neutrophils ameliorated clinical disease and reduced mycobacterial burden. However, neutrophils were not the critical source of itaconate in this model, because the cell type-specific deletion of ACOD1 in neutrophils (mediated by Mrp8-Cre) did not phenocopy the complete knockout, but deletion in all myeloid cells by LysM-Cre did (78).

In a model of traumatic tendon injury, itaconate production was shown to promote neutrophilia by increasing granulocyte-monocyte progenitor (GMP) output from the bone marrow (79). Interestingly, neutrophils present at the injury site were the major producers of itaconate. Still, exogenous administration of itaconate reduced inflammation and promoted resolution of inflammation, suggesting that also in neutrophils itaconate production may act as molecular brake to prevent exaggerated inflammation (79). An inhibitory effect of 4-OI on neutrophil formation of extracellular traps (NETs) has recently been described by Burczyk et al., associated with inhibition of HIF1α (80). The anti-microbial activity of neutrophils can be negatively affected by the high-level production of itaconate after *Staphylococcus aureus* infection *via* inhibition of the oxidative burst (30), thereby protecting tissues from immunopathology but interfering with bacterial clearance.

Expression of ACOD1 in neutrophils was shown to be induced by GM-CSF through the transcription factor C/EBPβ and to mediate resistance to ferroptosis via upregulation of the Nrf2-dependent anti-oxidant response genes *Gpx4*, *Gclc* and *Nqo1* (31). Thereby, survival of tumor-infiltrating neutrophils in a model of metastatic breast cancer was increased, which caused negative effects on anti-tumor T cell immunity (31).

### 4.2 Adaptive immune cells

The question whether itaconate production also impacts on adaptive immune responses only recently emerged in the literature. Although one study on human CD4^+^ T cells stimulated with an agonistic TNFR2 antibody reported secretion of itaconate into culture supernatants (81), to date the general notion that ACOD1 expression and generation of itaconate is restricted to myeloid cells remains unchanged. However, the clear demonstration that itaconate is released from macrophages and neutrophils into the extracellular space and can act in a paracrine or maybe even systemic manner on other cells (**Figure 2**), opened the scope of investigations toward other cells types as targets of endogenous itaconate. Moreover, exogenous application of cell permeable itaconate derivatives like DMI and 4-OI *per se* have the capacity to act on non-myeloid cells.

Following infection of *Acod1^-/-^* mice with an attenuated vaccine strain of *Francisella tularensis*, an increased bacterial burden and delayed clearance from lungs and spleen was observed (82), consistent with the reported role of endogenous itaconate in the control of *F. tularensis* in macrophages through inhibition of mitochondrial complex II (14). At the same time, *Acod1^-/-^* mice developed stronger pulmonary resident and splenic CD4^+^ and CD8^+^ T cell responses (82), that were associated with better protection against challenge with a virulent *F. tularensis* strain. T cells from vaccinated *Acod1^-/-^* mice enabled infected macrophages to better control bacterial growth *in vitro*. In contrast, addition of itaconate to T cell cultures did not affect their effector function although they took up itaconate intracellularly (82). Together, the results suggested an indirect enhancement of ACOD1-deficiency on the generation of protective T cell immunity through increased antigen exposure (higher peak bacterial burden and delayed clearance) or increased activation and function of antigen-presenting cells.

In a house dust mite model of Th2-dependent asthma induction in mice, Jaiswal et al. reported enhanced expansion of CD4^+^ T cells and differentiation to Th2 cells (38). *Acod1^-/-^* bone marrow-derived DC showed increased APC function *in vitro* and promoted increased generation of IL-4^+^ and IL-5^+^ Th2 cells. Exogenous 4-OI inhibited mitochondrial superoxide production by DC and, when applied intranasally in the mice, reduced Th2 cell and eosinophil responses (38). Thus, endogenous itaconate indirectly restrained antigen-specific Th2 responses *via* control of APC function.

In contrast, other studies also reported direct regulation of Th cell differentiation by itaconate and its derivatives. Aso et al. observed in the experimental allergic encephalomyelitis (EAE) model that treatment with itaconate *in vivo* reduced disease severity. Itaconate inhibited Th17 differentiation *in vitro* by suppressing glycolysis and oxidative phosphorylation, causing altered chromatin accessibility and decreased RORγt binding at the *Il17a* promoter (83). In contrast, Nastasi et al. recently reported that DMI and 4-OI, but not itaconate, inhibited human T cell activation and proliferation. Exogenous itaconate was taken up by activated CD4^+^ T cells but did not inhibit SDH activity as assessed by succinate levels (83).

The expression of ACOD1 in tumor-associated macrophages and the resulting production of itaconate and its release in the tumor microenvironment can inhibit T cell anti-tumor immune responses, as indicated by several studies. First, Zhao et al. showed that myeloid derived suppressor cells (MDSC) secrete itaconate that is taken up by CD8^+^ T cells, leading to their functional inactivation (37). *Acod1^-/-^* mice exhibited enhanced CD8^+^ T cell responses towards transplanted tumors, decreased MDSC accumulation and reduced tumor growth (37). Similar findings were reported very recently regarding a reduced tumor growth in *Acod1^-/-^* mice, that was associated with a reduced frequency of MDSC but increased T cell accumulation in the tumor microenvironment (84). *Acod1^-/-^* monocytes/macrophages promoted T cell trafficking in a Tet2-dependent manner and were sufficient in an adoptive transfer model to inhibit pancreatic tumor cell growth, again associated with enhanced CD8^+^ T cell influx (84).

Modulation of the itaconate pathway has also been investigated as a potential treatment strategy in autoimmune disease, specifically in a mouse model of systemic lupus erythematosus (SLE) (85). Lupus-prone NZW x NZB mice were treated with 4-OI administered *via* a subcutaneously implanted osmotic pump for 4 weeks. Systemic inflammation, production of autoantibodies and organ damage in the kidney were significantly reduced in 4-OI-treated mice. These effects correlated with an inhibition of expression of type I IFN and MAVS genes, and a reduction of glycolysis as well as OCR in splenocytes of 4-OI treated mice (85). Thus, although the mechanistic link between inhibition between the administration of 4-OI and the reduction in pathogenic autoantibodies is not yet clear, the itaconate pathway represents a target to modulate dysregulation of autoreactive B cell responses.

## 5 Conclusion and open questions

Since the identification of *Acod1*/*Irg1*-encoded ACOD1 as the enzyme responsible for generation of itaconate and its description as anti-microbial metabolite, we have seen a remarkable wealth of research findings on the functional role of ACOD1-itaconate in the immune system. The Janus-faced biology of itaconate as immunoregulatory and anti-microbial molecule has attracted the attention of infection immunologists as well as those working on the deleterious consequences of overshooting innate immune activation in sepsis and non-resolving inflammation. The search for the mechanistic basis of the immunoregulation by itaconate has been highly successful, maybe too successful, in that a plethora of molecular effects on metabolic enzymes, key signaling proteins and transcription factors have been revealed. The identification of itaconation, and recently of itaconylation, as post-translational cysteine (and lysine) modification affecting hundreds to thousands of cellular proteins provided a biochemical basis for many of itaconate’s effects.

The intention to utilize the newly discovered immunoregulatory properties of endogenous ACOD1-derived itaconate in a therapeutic approach led to the introduction of synthetic cell-permeable derivatives, 4-OI and DMI. While these clearly have many striking effects in models of infection, inflammation and related disease-conditions, their biological activity is not identical to that of itaconate because of changes in electrophilicity and potential different molecular interactions. Thus, the field has become complex by the need to separate the phenotypes caused by endogenous itaconate, exogenous itaconate and the synthetic derivatives.

Another complicating and highly interesting factor in ACOD1-itaconate biology is the species-difference in the capacity of mouse and human macrophages to produce itaconate. Intracellular levels of 5-10 mM in activated murine macrophages contrast with much lower (around 50-fold) itaconate concentrations in human macrophages (16, 45, 75). While recent publications also report stronger itaconate production from human macrophages (86), the demonstration of lower enzymatic activity of human ACOD1 compared to the murine protein provides an explanation for the species difference (87). It will be important to investigate more thoroughly itaconate levels in human cells and tissues. If the human system is indeed hypomorphic in itaconate production, the interpretation of studies obtained in *Acod1^-/-^* mice with regard to the human system needs to be done with particular caution. On the other hand, the effects of exogenous provision of itaconate (or its derivatives) may be considerably stronger in humans compared to mice that already produce ample itaconate themselves.

The intracellular trafficking, the release into the extracellular space and the uptake of itaconate by non-producing cells is an emerging and exciting topic in the field. The transport out of the mitochondria, where itaconate is produced, and trafficking to a pathogen-containing vacuole are partially understood (11, 35, 36). However, how different cells take up itaconate from the environment in changing activation states is currently completely unresolved. In the case of macrophages, it is now clear that exogenous itaconate, and its isomers, are readily taken up. It will be very interesting to learn in the future how these processes are mediated and regulated in different innate immune cells, in B and T cells, as well as in epithelial cells.

## Author contributions

RL: Conceptualization, Writing – original draft. MS: Writing – review and editing, Visualization.
